# Prenatal Exposure to SARS-Cov-2 and Schizophrenia Development: What to Expect?

**DOI:** 10.1192/j.eurpsy.2022.1338

**Published:** 2022-09-01

**Authors:** F. Tavares, M. Viseu, M. Barbosa Pinto, C. Solana

**Affiliations:** 1 University Hospital Center of Algarve, Portugal, Department Of Psychiatry And Mental Health – Faro, Faro, Portugal; 2 University Hospital Center of Algarve, Portugal, Department Of Psychiatry And Mental Health - Faro, Faro, Portugal

**Keywords:** Prenatal, Viral infection, schizophrénia, Covid-19

## Abstract

**Introduction:**

Schizophrenia is a complex and multifactorial psychiatric condition characterized by thought, speech, perception and behaviour disorders, and social and occupational impairment. It has been related that viral prenatal infection may contribute to schizophrenia development. As such, there are some hypotheses regarding SARS-Cov-2 prenatal infection and its potential relation with “future” offspring schizophrenia.

**Objectives:**

Literature review of schizophrenia development and relation with viral infections, and data research of COVID-19 neurotropic effects.

**Methods:**

Non-systematic review through literature using databases as Pubmed and UpToDate. Keywords used: schizophrenia, prenatal, viral infection, COVID-19, SARS-Cov-2.

**Results:**

Several studies had shown a relationship between prenatal viral infections, such as Influenza, and development of schizophrenia in the offspring. It relates with viral neurotropism mechanisms and inflammatory processes in the fetal neurology system. Regarding SARS-Cov-2, it is early to assume a relation between prenatal COVID-19 and offspring schizophrenia development. However, literature describes psychiatric manifestations post COVID, such as psychotic and manic episodes. As such, a SARS-Cov-2 neurotropic effect is demonstrated.

**Conclusions:**

Schizophrenia has a multifactorial etiology. Since prenatal viral infections may interfere and contribute to schizophrenia development, it is logical to assume prenatal SARS-Cov-2 infection may also contribute. It may be relevant to investigate whether these offspring will manifest schizophrenia symptoms.

**Disclosure:**

No significant relationships.

